# Acceptance of Leadership Empowerment Efforts for Female Employees in Three Dental Hospitals

**DOI:** 10.1155/2020/8056175

**Published:** 2020-08-03

**Authors:** Tadeus Arufan Jasrin, Anne Agustina Suwargiani, Riana Wardani, Dudi Aripin, Franky Oscar, Andi Supriatna, Inne S. Sasmita

**Affiliations:** ^1^Department of Oral Biology, Faculty of Dentistry, Universitas Padjadjaran, Sumedang, Indonesia; ^2^Department of Dental Public Health, Faculty of Dentistry, Universitas Padjadjaran, Sumedang, Indonesia; ^3^Department of Conservative Dentistry, Faculty of Dentistry, Universitas Padjadjaran, Sumedang, Indonesia; ^4^Department of Oral and Maxillofacial Surgery, Faculty of Dentistry, Maranatha Christian University, Bandung, Indonesia; ^5^Department of Dental Public Health, Faculty of Medicine, Jenderal Achmad Yani University, Kota Cimahi, Indonesia; ^6^Department of Pediatric Dentistry, Faculty of Dentistry, Universitas Padjadjaran, Sumedang, Indonesia

## Abstract

**Introduction:**

Empowerment is an organisational effort in increasing the willingness and ability of employees to be more independent in performing their duties. Employee leadership empowerment substantially increases motivation, performance, and commitment to the organisation. Leadership empowerment includes self-determination, competence, meaning, and impact. The purpose of this study was to determine the acceptance of leadership empowerment efforts for female employees in three dental hospitals in Bandung.

**Methods:**

A descriptive survey was conducted and the study population was dental hospitals in Bandung. The exclusion criteria were dental hospitals which unwillingly followed the research. The inclusion criteria were dental hospitals that have empowered female employees. The data collection technique was performed with total sampling. The research sample amounted to 102 people from three dental hospitals—all data were collected using a validated questionnaire. Data analysis was performed using relative frequency distribution.

**Results:**

Leadership empowerment for female employees in three dental hospitals in Bandung has been given by each. The highest acceptance of empowerment efforts for female employees was shown by employees of Dental Hospital of Jenderal Achmad Yani University, followed by Dental Hospital of Universitas Padjadjaran, and subsequently Dental Hospital of Maranatha Christian University. The level of acceptance of empowerment efforts that need to be improved in three Dental Hospitals in Bandung was self-determination or autonomy at work. Employees must be given more freedom on performing their duties, developing their careers, and acting more independently.

**Conclusion:**

Acceptance of leadership empowerment for female employees in three dental hospitals in Bandung has been demonstrated in each. Acceptance of leadership empowerment efforts in all subdimensions of empowerment needs to be improved, primarily related to self-determination.

## 1. Introduction

Some previous researchers have argued that such an environment affects these women's ability to fulfil their family needs. Besides, impoverished women hold responsibility and contribute towards the survival of their impoverished households. Other studies about women and impoverished households also show the importance of women's role as the primary wage earner in their household. Moreover, the result of the study conducted in East Java showed that impoverished women did various works, with a majority of those working in the informal sectors for more than 8 hours per day and earning relatively small wages [1].

Employee empowerment in the form of employee involvement and commitment is a significant success factor in the current organisation. Empowerment increases employees' personal sense of control and motivates them to be more engaged in work, which in turn produces positive managerial and organisational results [1]. Employee involvement as an “engine” of managerial actions will attract the participation of various environmental factors from inside and outside of the organisation effectively. Initiatives originating from employee involvement are very strategic in supporting the branding and reputation of the organisation [2].

The empowerment approach for women must be performed through a gender empowerment approach. Gender empowerment should not be misinterpreted as empowering men against women vice versa. Empowerment signifies the transformation of gender relations from a planned sequence of levels or ranks to equality rather than just fiddling with women's positions of power [3].

The next approach is the “Efficiency of Gender Perspective” that focuses on the efficiency of women's role, with an assumption that bringing women into production will automatically serve as a means of achieving gender equality. According to this efficiency approach, the development will only be successful and efficient if women are involved in the development process [1]. However, the assumption that the women's participation in the economy may increase gender equality has been widely criticised as some factors hamper women's participation, such as having low levels of education and lacking knowledge in technology. Even though the role of women is essential for the success of economic development, their role in development does not automatically [1].

Increase the status of women to achieve gender equality. The results showed that empowerment, which includes four dimensions of potential, meaningfulness, autonomy, and impact, is more likely to improve organisational performance compared to traditional leadership. The improvement was mainly reflected in employee performance results such as team productivity, proactive quality, and customer service, also towards employee attitudes such as job satisfaction and organisational commitment [4].

Empowering women is beneficial because it can shape behaviour towards and from the organisation. The benefits are as follows: (a) personal: develop confidence and self-capacity and eliminate internalised oppression; (b) rational: develop the ability to negotiate and influence the nature of relationships and decisions made therein; and (c) collective.

Empowerment through increased competence, work meaningfulness, autonomy, and impact is beneficial to improve work team efficiency, thus impacts on the performance and working attitude. Implications on performance are increasing productivity, proactivity, and customer service, while on the working attitudes are increasing job satisfaction and organisational commitment. This importance of empowerment encourages us to investigate the acceptance of empowerment in dental hospitals in Bandung. This study was aimed to determine the acceptance of leadership empowerment efforts for female employees in three dental hospitals in Bandung.

## 2. Methods

The research was a descriptive survey [5]. The study population was the three dental hospitals located. The sampling technique used was the total sampling in the population of West Java government office and a private company and resulted in three dental hospitals that were taken as the sample. The exclusion criteria were dental hospitals which unwillingly followed the research. The inclusion criteria were dental hospitals that have empowered female employees.

The data collection technique used was the survey technique. The number of research samples that met the inclusion criteria was at a total of 102 people. The data collection technique was carried out using a questionnaire. Empowerment questionnaires in this study included self-determinants/freedoms, meaningfulness/value of the work done, competence/ability to work, and the impact of self-existence. The research instrument was created by the authors. The validity and reliability of the questionnaire were the results of the validity test in the form of a correlation coefficient above 0.4 (strong correlation) and some that reach above 0.7 (very strong correlation). The reliability test results were in the form of Cronbach's alpha value of 0.842. This value was reliable because it had the value above 0.7 [6].

Scale of data acceptance was calculated using the Likert scale with five categories, which were strongly agree, agree, neither agree, disagree, and strongly disagree. Data analysis was performed using relative frequency distribution in Microsoft® Excel. The study was conducted at the Dental Hospital of Maranatha Christian University, Dental Hospital of Jenderal Achmad Yani University, and Dental Hospital of Universitas Padjadjaran by following the research ethics requirements required by the Research Ethics Committee of Universitas Padjadjaran.

## 3. Results

The results of the current research are presented in Table 1, which shows that all empowerment has acceptance at Universitas Padjadjaran Dental Hospital. The high acceptances in the empowerment are competence and impact. Acceptances in the empowerment of competence are believe I can do all of my tasks and want to learn and be able to solve new challenges at work well and on time. Acceptances in the empowerment of impact are all decisions and actions are always taken into consideration of the progress of the dental hospital.

Table 2 shows that all empowerment has acceptance at Jenderal Achmad Yani University Dental Hospital. The high acceptances in the empowerment are meaningful and impact. Acceptances in the empowerment of meaningful are believe concerned about the quality of my work and the work is needed by other departments and the entire organisation. Acceptances in the empowerment of impact are all decisions and actions are always taken into consideration of the progress of the dental hospital.

Table 3 shows that all empowerment has acceptance at Maranatha Christian University Dental Hospital. The high empowerment effort accepted was the competence regarding the employee's belief that they can do all of their tasks.

The three tables above show that all three dental hospitals in Bandung have provided leadership empowerment for female employees, with acceptance of the empowerment effort shown variation in the value.

Table 4 describes the significance value of the normality test *p* value was in the range of 0.000–0.007, which was lower than 0.05. This value indicated that the variable data above were not normally distributed.

Table 5 shows the rank value based on the mean for Q1 of self-determination: having the freedom to run and convey ideas within the organisation in Universitas Padjadjaran Dental Hospital was the highest, followed by Maranatha and Jenderal Achmad Yani University Dental Hospital. However, regarding the Q2 of self-determination: having the freedom to determine career development, Maranatha Dental Hospital was found of having the highest, followed by Universitas Padjadjaran and Jenderal Achmad Yani University Dental Hospitals. In the Q3 of self-determination: set the policy independently for duties in the organisation, Maranatha Dental Hospital was also had the highest value.

The rank value based on the mean for Q1 of meaning: concerned about the quality of my work, Jenderal Achmad Yani University Dental Hospital had the highest value, followed by Universitas Padjadjaran and Maranatha. Regarding the Q2 of meaning: the work is needed by other departments and the entire organisation, Jenderal Achmad Yani University Dental Hospital also had the highest value, followed by Universitas Padjadjaran and Maranatha.

The rank value based on the mean for Q1 of competence: the belief that I can do all of my tasks, Jenderal Achmad Yani University Dental Hospital had the highest value, followed by Maranatha and Universitas Padjadjaran, and also, regarding the Q2 of competence: want to learn and be able to solve new challenges at work well and on time, Jenderal Achmad Yani University Dental Hospital also had a slightly higher value than Universitas Padjadjaran and Maranatha Dental Hospital.

The rank value based on the mean for the Q1 of impact: actively engaged in all assignments for the advancement of the Dental Hospital organisation, Jenderal Achmad Yani University Dental Hospital had the highest value, followed by Universitas Padjadjaran and Maranatha, and also, regarding the Q2 of impact: all decisions and actions that I take are always taken into consideration of the progress of the Dental Hospital, Jenderal Achmad Yani University Dental Hospital also had a slightly higher value than Universitas Padjadjaran and Maranatha Dental Hospital. All results above are described in Table 5.

Table 6 shows that all *p* values were higher than 0.05. These values indicated no significant difference in the acceptance of empowerment efforts for female employees in three dental hospitals.

## 4. Discussion

Tables 1–3 suggest that the acceptance of leadership empowerment for female employees that need to be improved in the three dental hospitals was regarding the self-determination or freedom to work, where employees must be given more freedom to perform their duties, career development, and independent policy. These results were in line from the results of problems in the implementation of women empowerment activities organized by government or community institutions indicate that the number of women is large enough, yet cannot be utilized all the potential for development purposes because women cannot enjoy life better because of the shackles of cultural sanctions. The theoretical studies developed related to building women through entrepreneurship programs are one of the reasons to empower women to be able to create entrepreneurship opportunities [7]. Empirical analysis based on the 2010 Federal Employee Viewpoint Survey (FEVS) data showed that the practice of empowerment aimed to promote self-determination has a positive and considerable effect [8].

The results of the current research indicated the necessity for a leadership empowerment approach for female employees, where this effort will have a positive effect on job satisfaction in the form of increasing self-determination. Sharing information regarding goals and performance, providing access to work-related knowledge and skills, and providing wisdom to alter the work processes have a positive and appreciable effect on job satisfaction. Conversely, empowering practices that undermine autonomy, such as offering contingent-based rewards, do not have any significant influence on the job satisfaction [8].

Self-determination is a valuable part of empowerment because it includes the concept of individual autonomy, good behavior competence, and the ability to assess the relationship between behavior and its results [9]. Self-determination is formed from two forms of motivation: intrinsic motivation, which is related to pleasure and excitement, and extrinsic motivation, which is related to the reasons for achieving personal goals [10].

Self-determination theory indicates that every individual has an innate tendency towards future development and intrinsic motivation. Intrinsic motivation and well-being are part of three psychological needs related to competence and autonomy. Social rules, including rules at work, external rules, or stimuli including payments, supervision, goals, and direction, are used to encourage desirable behaviour [9].

As long as these three needs are fulfilled, employees will internalize and integrate external rules, meaning they will take values and accept these external rules as their values and self-motivation. This process of internalization and integration forms extrinsically determined behaviors and internal motivation to act on their own accord [8].

Tables 1–3 show that all employees in three dental hospitals agree that they pay attention to the quality of work and agree that the organisation needs the job they perform. These results were consistent with research regarding the meaningfulness of work that illustrates not only the significant and positive contribution to the meaningfulness of an individual life, but also to enjoying work, which implies to the variety of cognitive, emotional, behavioral, and economic benefits. The meaningfulness in work is defined as the importance of working goals in people's views regarding how their lives and attitudes can be related to variables in the area of organisational behavior. This term is also defined as the importance of working goals in an individual view of life and attitude [11].

People have many definitions of work at the same time. However, it usually seems to dominate at specific periods. To be able to see how the meaning of work is built up over time, it is essential to classify people depending on the meaning of work that is dominant to them. Two categorizations were made based on experience. The meaning/function of the main job is to meet the social and economic expectations that are mostly asked by members of their family and relevant community. The meaning of work is considered to be “social” when the meaning/function of the main job is to meet individual needs, aspirations, and interests, and thus, the job can also be considered as having a “personal” meaning. As stated, these categories are not exclusive to each other, and an individual can have both meanings at a certain point. The process of how one meaning dominates another is the subject matter being explored [12].

Table 1 shows leadership empowerment that needs to be improved at Universitas Padjadjaran Dental Hospital is active engagement in all assignments for the advancement of the organisation, through liberating the female employees to express their views on decisions that might affect their functions. Leadership from a very active workplace creates a more challenging and trustworthy environment, where female employees will be able to innovate and help organizations grow. The opportunity of employees on expressing their views to the senior management will have an impact on engagement participation [3].

Such practices also the form of joint control that rewards, recognizes, and values the employee's behavior that can predict employee involvement while providing a higher commitment to their superiors and increasing the level of involvement or participation, which leads to higher learning efforts that will enhance innovation in the workplace. Employees will feel more empowered when they feel that their manager is an empowering leader, which in turn gives motivation and a sense of belonging to the organisation and leads them to be more engaged [3]. The results of the current research, however, were different from what has been stated by Anuradha et al. [12]; social expectations and fulfilling the roles and responsibilities are not the primary determinants of the meaning of work. Even before entering the job, the work for several people is seen as a means of expressing someone's abilities and interests. This definition has been called the “personal” meaning of work because work is seen as a means of meeting individual needs rather than as a means of fulfilling social roles and responsibilities [12].

Table 2 shows the opinions of employees regarding empowerment that need to be improved at Jenderal Achmad Yani University Dental Hospital. The participation of employees needs to be improved for the advancement of the hospital. Increasing employee participation must be performed by first analyzing their roles.

Employee involvement consists of three different levels, which are the employee involved, not involved, and unable to get involved. Employees involved are those who work with enthusiasm towards the organisation goals. Employees who are not involved are employees who seem to be participating but not with enthusiasm and energy for achieving the organisation's shared goals. Employees who are unable to get involved are people who are unhappy at work, because of an act of their unhappiness [3].

Involvement also has three different aspects, namely, intellectual involvement, which refers to a dedication to perform better action; effective involvement, which is a positive feeling when performing the job; and social involvement, which is showed through discussing with others regarding improvements in performance related to their work [3].

Efforts to increase the role can be done with career development. Organizations with highly engaged employees will provide many opportunities to learn skills, develop abilities, gain knowledge, and reach their potential. Career development practices will help organizations to retain talented employees and also provide personal development opportunities [3].

Organizations can invest in employees by planning their career development. Career development is a crucial factor in involving employees. Adequate employee career development through training, skills, and learning can result in employees that are more committed to their work and organisation [3].

All three dental hospitals in Bandung have provided leadership empowerment for their female employees. Empowerment access has been recognized by their female employees, which expressed in terms of strongly agree and agree. The most attitude scale stated by employees who have gained access to empowerment is agreed.

Empowering women is an important goal in achieving sustainable development throughout the world. Offering access to microfinance services for women is one way to increase women's empowerment. However, empirical evidence gives mixed results concerning its effectiveness [13].

The three-dimensional model of women's empowerment has been developed to integrate previous findings and to gain a deeper understanding of women's empowerment. This model proposes that women's empowerment can occur in three different dimensions: (1) microlevel, referring to personal beliefs and actions, where personal empowerment can be observed; (2) mesolevel, referring to beliefs and actions concerning others relevant, where relational empowerment can be observed; and (3) macrolevel, referring to results in a broader social context where social empowerment can be observed [13].

The results of the current research presented in the three tables above can be intervened in the short term. Previous interventions have proven to be successful in increasing women's incomes and thus should not be underestimated. These interventions benefit women and can have a transformative impact on society by encouraging more significant investment in children welfare, reducing household poverty, and increasing aspirations for the next generation of women and girls [14].

Interventions that can be conducted in the short term, based on the results of previous research, are providing women with business management training, workplace and skills training, and also job vouchers. These are increasingly common interventions aimed at increasing the productivity and income of women entrepreneurs, women farmers, and female wage workers in developing countries [14].

The results of previous studies confirmed significant differences in empowerment and job satisfaction based on demographic factors, which are gender, age, marital status, educational qualifications, appointment, income, and experience. Age, qualifications, and educational background have significant differences in empowerment [15]. Women's empowerment and gender equality are interrelated concepts. However, these two concepts must be emphasized to improve job satisfaction and consequently, the performance of female employees [16].

The results of the present study indicated that an increase in employee empowerment is an urgent need because empowerment is the type of motivational strategy that gives employees a sense of satisfaction with their work and organisation. It can be concluded that employees were satisfied with various factors such as employee training, self-development programs, employee meetings, employee participation in multiple activities, and consideration of their ideas and opinions [17, 18].

Overall, there were no significant differences in the acceptance of empowerment efforts for female employees in three dental hospitals. This result may be caused by each hospital that has advantages in several types of empowerment and has limitations in several types of empowerment as listed in Table 5. Table 5 also shows empowerment in the three dental hospitals must be empowered because the mean is in the range of 41.35–59.58. Acceptance of leadership empowerment for female employees in three dental hospitals in Bandung did not differ. Acceptance of leadership empowerment efforts in all subdimensions of empowerment needs to be improved because this is important to motivate women and referring to results in a broader social context.

## 5. Conclusion

Leadership empowerment in three dental hospitals has been implemented and accepted (recognized) by female employees because of their perceived benefits. Acceptance of leadership empowerment efforts in all subdimensions of empowerment needs to be improved.

## Figures and Tables

**Table 1:** Acceptance of leadership empowerment efforts at Universitas Padjadjaran Dental Hospital.

No.	Dimension	Strongly agree		Agree		Neither agree		Disagree		Strongly disagree		Total *N*
		*N*	%	*N*	%	*N*	%	*N*	%	*N*	%	
1	Self-determination											
	Have the freedom to run and convey ideas within the organisation	3	7.7	14	35.9	14	35.9	5	12.8	3	7.7	39
	Have the freedom to determine career development (Q2)	4	10.3	12	30.8	15	38.5	6	15.4	2	5.1	39
	Set the policy independently for duties in the organisation (Q3)	2	5.1	10	25.6	17	43.6	7	17.9	3	7.7	39

2	Meaningfulness											
	Concerned about the quality of my work (Q1)	10	25.6	25	64.1	4	10.3	0	0.0	0	0.0	39
	The work is needed by other departments and the entire organisation (Q2)	6	15.4	25	64.1	5	12.8	3	7.7	0	0.0	39

3	Competence											
	Believe I can do all of my tasks	11	28.2	26	66.7	1	2.6	1	2.6	0	0.0	39
	Want to learn and be able to solve new challenges at work well and on time	8	20.5	26	66.7	5	12.8	0	0.0	0	0.0	39

4	Impact											
	Actively engage in all assignments for the advancement of the dental hospital organisation	2	5.1	23	59.0	13	33.3	1	2.6	0	0.0	39
	All decisions and actions are always taken into consideration of the progress of the dental hospital	5	12.8	26	66.7	8	20.5	0	0.0	0	0.0	39

**Table 2:** Acceptance of leadership empowerment efforts at Jenderal Achmad Yani University Dental Hospital.

No.	Dimension	Strongly agree		Agree		Neither agree		Disagree		Strongly disagree		Total *N*
		*N*	%	*N*	%	*N*	%	*N*	%	*N*	%	
1	Self-determination											
	Have the freedom to run and convey ideas within the organisation (Q1)	1	3.8	18	69.2	4	15.4	3	11.5	0	0.0	26
	Have the freedom to determine career development	0	0.0	17	65.4	6	23.1	3	11.5	0	0.0	26
	Set the policy independently for my duties in the organisation	2	7.7	13	50.0	6	23.1	4	15.4	1	3.8	26

2	Meaningfulness											
	Concerned about the quality of my work	2	7.7	20	76.9	4	15.4	0	0.0	0	0.0	26
	The work is needed by other departments and the entire organisation	4	15.4	12	46.2	8	30.8	1	3.8	1	3.8	26

3	Competence											
	Believe I can do all of my tasks	5	19.2	17	65.4	4	15.4	0	0.0	0	0.0	26
	Want to learn and be able to solve new challenges at work well and on time	5	19.2	18	69.2	3	11.5	0	0.0	0	0.0	26

4	Impact											
	Actively engage in all assignments for the advancement of the dental hospital organisation	0	0.0	18	69.2	7	26.9	1	3.8	0	0.0	26
	All decisions and actions that I take are always taken into consideration of the progress of the dental hospital	0	0.0	20	76.9	5	19.2	1	3.8	0	0.0	26

**Table 3:** Acceptance of leadership empowerment efforts at Maranatha Christian University Dental Hospital.

No.	Dimension	Strongly agree		Agree		Neither agree		Disagree		Strongly disagree		Total *N*
		*N*	%	*N*	%	*N*	%	*N*	%	*N*	%	
1	Self-determination											
	Have the freedom to run and convey ideas within the organisation (Q1)	6	16.2	16	43.2	10	27.0	4	10.8	1	2.7	37
	Have the freedom to determine career development (Q2)	3	8.1	11	29.7	10	27.0	10	27.0	3	8.1	37
	Set the policy independently for my duties in the organisation (Q3)	3	8.1	10	27.0	12	32.4	8	21.6	4	10.8	37

2	Meaningfulness											
	Very concerned about the quality of my work (Q1)	12	32.4	20	54.1	5	13.5	0	0.0	0	0.0	37
	The work is needed by other departments and the entire organisation (Q2)	14	37.8	17	45.9	5	13.5	1	2.7	0	0.0	37

3	Competence											
	Believe I can do all of my tasks (Q1)	9	24.3	23	62.2	4	10.8	1	2.7	0	0.0	37
	Want to learn and be able to solve new challenges at work well and on time (Q2)	11	29.7	19	51.4	6	16.2	1	2.7	0	0.0	37

4	Impact											
	Actively engage in all assignments for the advancement of the dental hospital organisation (Q1)	12	32.4	15	40.5	9	24.3	1	2.7	0	0.0	37
	All decisions and actions that I take are always taken into consideration of the progress of the dental hospital (Q2)	11	29.7	18	48.6	7	18.9	0	0.0	1	2.7	37

**Table 4:** Shapiro–Wilk normality test results.

Subvariable	Dental hospital code	Statistic	df	Sig.
Q1 self-determination	1.00	0.888	37	0.001
	2.00	0.702	26	0.000
	3.00	0.895	39	0.002

Q2 self-determination	1.00	0.914	37	0.007
	2.00	0.667	26	0.000
	3.00	0.911	39	0.004

Q3 self-determination	1.00	0.919	37	0.010
	2.00	0.862	26	0.002
	3.00	0.904	39	0.003

Q1 meaning	1.00	0.788	37	0.000
	2.00	0.657	26	0.000
	3.00	0.751	39	0.000

Q2 meaning	1.00	0.785	37	0.000
	2.00	0.864	26	0.003
	3.00	0.777	39	0.000

Q1 competence	1.00	0.782	37	0.000
	2.00	0.762	26	0.000
	3.00	0.695	39	0.000

Q2 competence	1.00	0.828	37	0.000
	2.00	0.733	26	0.000
	3.00	0.747	39	0.000

Q1 impact	1.00	0.844	37	0.000
	2.00	0.633	26	0.000
	3.00	0.780	39	0.000

Q2 impact	1.00	0.809	37	0.000
	2.00	0.558	26	0.000
	3.00	0.747	39	0.000

**Table 5:** Kruskal–Wallis rank test results.

Subvariable	Dental hospital code	*N*	Mean rank
Q1 self-determination	1.00	37	48.12
	2.00	26	45.92
	3.00	39	58.42
	Total	102	

Q2 self-determination	1.00	37	57.42
	2.00	26	42.67
	3.00	39	51.77
	Total	102	

Q3 self-determination	1.00	37	54.65
	2.00	26	42.56
	3.00	39	54.47
	Total	102	

Q1 meaning	1.00	37	47.84
	2.00	26	59.58
	3.00	39	49.59
	Total	102	

Q2 meaning	1.00	37	41.35
	2.00	26	61.21
	3.00	39	54.65
	Total	102	

Q1 competence	1.00	37	52.65
	2.00	26	55.52
	3.00	39	47.73
	Total	102	

Q2 competence	1.00	37	50.55
	2.00	26	52.08
	3.00	39	52.01
	Total	102	

Q1 impact	1.00	37	43.07
	2.00	26	56.19
	3.00	39	56.37
	Total	102	

Q2 impact	1.00	37	46.00
	2.00	26	58.60
	3.00	39	51.99
	Total	102	

**Table 6:** Kruskal–Wallis test results of the acceptance of empowerment efforts for female employees in three dental hospitals.

Subvariable	Kruskal–Wallis	df	Sig.
Q1 self-determination	4.058	2	0.131
Q2 self-determination	4.197	2	0.123
Q3 self-determination	3.470	2	0.176
Q1 meaning	3.672	2	0.159
Q2 meaning	9.137	2	0.010
Q1 competence	1.637	2	0.441
Q2 competence	0.79	2	0.961
Q1 impact	5.830	2	0.054
Q2 impact	3.754	2	0.153

## Data Availability

All data used for this research are publicly available and accessible online.
